# Physicians’ Visiting List

**Published:** 1880-12

**Authors:** 


### PHYSICIANS' VISITING LIST.

Lindsay & Blakiston have just issued The Physicians' Visiting List and Engagement Book for 1880.

This is a most valuable aid to the physician. It is prepared with special reference to his requirements, for appointments, records, and memorandums pertaining to daily practice. In addition to the blanks for these purposes, there are given the metric or French decimal system of weights and measures, posological tables, showing the relations of our present system of apothecaries' weights and measures to that of the metric system; gives the doses in both. There is certainly nothing better than this for the physicians' use, and I know of nothing that equals it in completeness in all respects.

It is prepared for twenty-five to one hundred patients, and is in price from $1 to $2, and may be had of the publishers or of any bookseller in the country.

